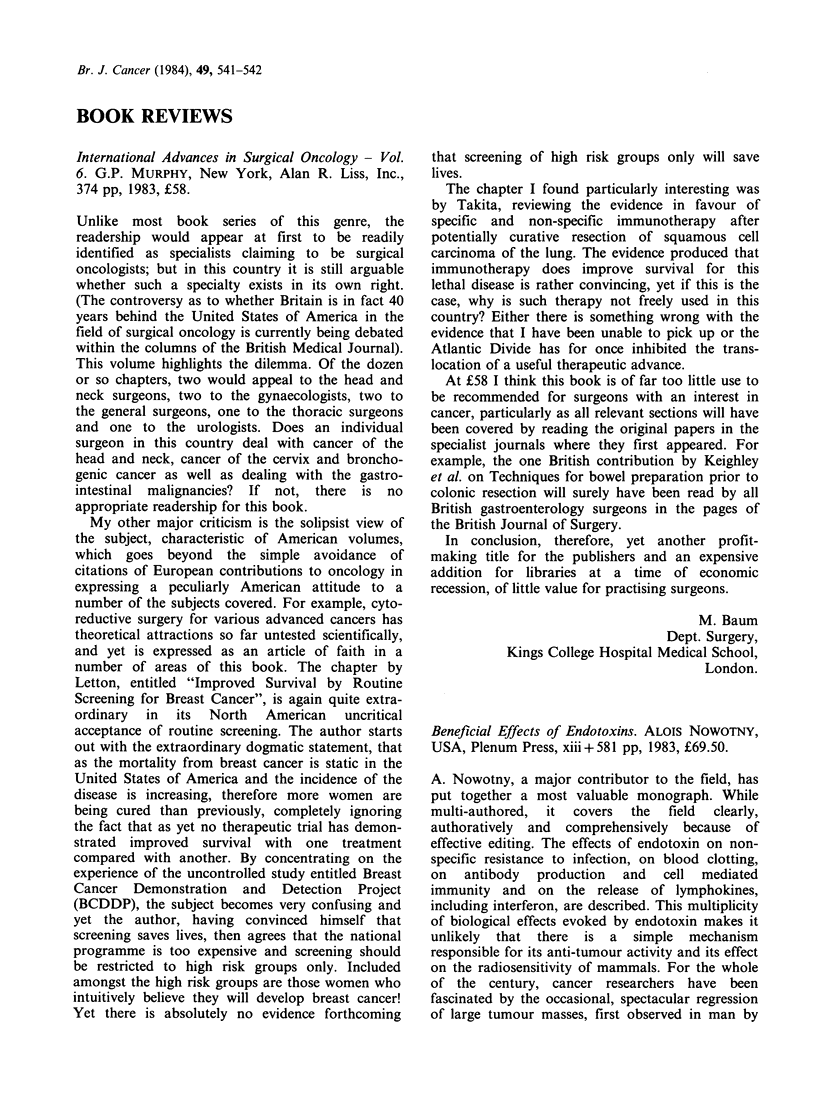# International Advances in Surgical Oncology - Vol. 6

**Published:** 1984-04

**Authors:** M. Baum


*Br. J. Cancer* (1984), **49**, 541–542

### BOOK REVIEWS

*International Advances in Surgical Oncology – Vol. 6.* G.P. MURPHY, New York, Alan R. Liss, Inc., 374 pp, 1983, £58.

Unlike most book series of this genre, the readership would appear at first to be readily identified as specialists claiming to be surgical oncologists; but in this country it is still arguable whether such a specialty exists in its own right. (The controversy as to whether Britain is in fact 40 years behind the United States of America in the field of surgical oncology is currently being debated within the columns of the British Medical Journal). This volume highlights the dilemma. Of the dozen or so chapters, two would appeal to the head and neck surgeons, two to the gynaecologists, two to the general surgeons, one to the thoracic surgeons and one to the urologists. Does an individual surgeon in this country deal with cancer of the head and neck, cancer of the cervix and bronchogenic cancer as well as dealing with the gastrointestinal malignancies? If not, there is no appropriate readership for this book.

My other major criticism is the solipsist view of the subject, characteristic of American volumes, which goes beyond the simple avoidance of citations of European contributions to oncology in expressing a peculiarly American attitude to a number of the subjects covered. For example, cytoreductive surgery for various advanced cancers has theoretical attractions so far untested scientifically, and yet is expressed as an article of faith in a number of areas of this book. The chapter by Letton, entitled "Improved Survival by Routine Screening for Breast Cancer", is again quite extraordinary in its North American uncritical acceptance of routine screening. The author starts out with the extraordinary dogmatic statement, that as the mortality from breast cancer is static in the United States of America and the incidence of the disease is increasing, therefore more women are being cured than previously, completely ignoring the fact that as yet no therapeutic trial has demonstrated improved survival with one treatment compared with another. By concentrating on the experience of the uncontrolled study entitled Breast Cancer Demonstration and Detection Project (BCDDP), the subject becomes very confusing and yet the author, having convinced himself that screening saves lives, then agrees that the national programme is too expensive and screening should be restricted to high risk groups only. Included amongst the high risk groups are those women who intuitively believe they will develop breast cancer! Yet there is absolutely no evidence forthcoming that screening of high risk groups only will save lives.

The chapter I found particularly interesting was by Takita, reviewing the evidence in favour of specific and non-specific immunotherapy after potentially curative resection of squamous cell carcinoma of the lung. The evidence produced that immunotherapy does improve survival for this lethal disease is rather convincing, yet if this is the case, why is such therapy not freely used in this country? Either there is something wrong with the evidence that I have been unable to pick up or the Atlantic Divide has for once inhibited the translocation of a useful therapeutic advance.

At £58 I think this book is of far too little use to be recommended for surgeons with an interest in cancer, particularly as all relevant sections will have been covered by reading the original papers in the specialist journals where they first appeared. For example, the one British contribution by Keighley *et al.* on Techniques for bowel preparation prior to colonic resection will surely have been read by all British gastroenterology surgeons in the pages of the British Journal of Surgery.

In conclusion, therefore, yet another profit-making title for the publishers and an expensive addition for libraries at a time of economic recession, of little value for practising surgeons.

M. Baum
Dept. Surgery,
Kings College Hospital Medical School,
London.